# CircASH1L-mediated tumor progression in triple-negative breast cancer: PI3K/AKT pathway mechanisms

**DOI:** 10.1515/med-2025-1261

**Published:** 2025-08-19

**Authors:** Haiyan Liu, Jiaming Wu, Jin Gong, Jing Zhu, Jie Liu, Xiaoqing Chen, Shaohua Qu, Jintao Chen, Zhisheng Zhou, Xulong Fan

**Affiliations:** Foshan Maternal and Child Health Hospital, Foshan, Guangdong, China; Department of Neurosurgery, The First Affiliated Hospital of Jinan University, Guangzhou, 510630, China; The First Affiliated Hospital of Jinan University, No. 613 Huangpu venue West, Tianhe District, Guangzhou, Guangdong, China; Department of Breast Surgery, The First Affiliated Hospital of Jinan University, Guangzhou, Guangdong, China; Foshan Maternal and Child Health Hospital, No. 11 Renmin West Road, Chancheng District, Foshan, Guangdong, China

**Keywords:** circASH1L, PI3K/AKT pathway, triple-negative breast cancer, tumor growth

## Abstract

**Objective:**

To investigate the impact of circASH1L on subcutaneous tumor growth in nude mice with triple-negative breast cancer via the PI3K/AKT pathway.

**Methods:**

The study was conducted using bioinformatics and animal experimental verification methods. circASH1L levels in triple-negative breast cancer (TNBC) were analyzed using a dataset from the gene expression omnibus database. In the animal experiment part, nude mice were divided into shNC group, shcircASH1L-1 group, Oe-NC group, and Oe-circASH1L group. Each group was treated with corresponding circASH1L overexpression or knockdown and transplanted tumor modeling. Immunohistochemistry and western blot experiments were used to verify the effect of circASH1L on the growth of nude mouse transplanted tumors and the PI3K/AKT pathway.

**Results:**

A total of 43 circRNAs were significantly associated with TNBC, among which circASH1L was significantly highly expressed in TNBC. circASH1L-1 negatively regulates tumor volume, mass, expression rate of Ki67 cells, and PI3K/AKT pathway marker proteins.

**Conclusions:**

CircASH1L is a tumor promoter in TNBC. The expression level of circASH1L influences both the proliferation of TNBC cells and the growth of TNBC nude mice tumors by modulating the PI3K/AKT pathway.

## 1 Introduction

Breast cancer ranks second among all causes of death among women worldwide, and the age of onset tends to be younger [1]. Triple-negative breast cancer (TNBC) accounts for about 15% of all breast cancer cases and has the poorest prognosis and highest mortality rate among the sub-types, approximately half of patients will relapse within 3–5 years [2,3]. HER2, ER, and PR expressions are absent in TNBC, and is ineffective for treatments that are usually effective for other sub types, such as hormone therapy, which makes the treatment of TNBC more difficult [4,5]. Early detection and early treatment are the key to effective treatment of TNBC. The early symptoms are non-specific, and X-ray imaging does not have typical features compared to other sub types [6,7]. TNBC is a highly invasive cancer that is often discovered in the late stage, delaying the best treatment time. Currently, chemotherapy is its main treatment method, but it has not yet achieved the desired effect. The current standard chemotherapy method is only effective for non-metastatic TNBC, and has limited treatment effects on metastatic and recurrent TNBC, and cannot achieve ideal therapeutic effects. Therefore, it is particularly important to seek early diagnosis targets for TNBC and new chemotherapy targets. TNBC has a relatively complex pathogenesis, and in recent decades, whole genome sequencing and multi-access studies such as protoplasmic have found that the pathogenesis of TNBC involves the complex interaction of multiple genetic molecules, and there is a high degree of molecular heterogeneity both within and between TNBC tumors. Therefore, it is very important to seek NBC-specific potential therapeutic targets. Currently, research on its related targets mainly focuses on non-coding RNAs such as marinas, pancreas, Micronesia, and circulars as well as linear or even network regulatory effects on each other [8–12]. Circulars, as a typical circular non-coding RNA, have emerged in the field of TNBC research in recent years. Currently, aircraft [13], circSEPT9 [14], circGFRA1 [15], circCD44 [16], etc. have been confirmed to be involved in the pathogenesis of TNBC. As a new target discovered in recent years, circASH1L has not been reported in other cancers including TNBC except cervical cancer [17]. The PI3K/AKT pathway is involved in the progression of multiple cancers, such as TNBC. Reports indicate that circadian influences the PI3K/AKT signaling pathway, contributing to breast cancer diagnosis and treatment [18]. The regulatory mechanism of circASH1L and the PI3K/AKT pathway in TNBC remains largely unexplored and warrants further investigation.

**Figure 1:**
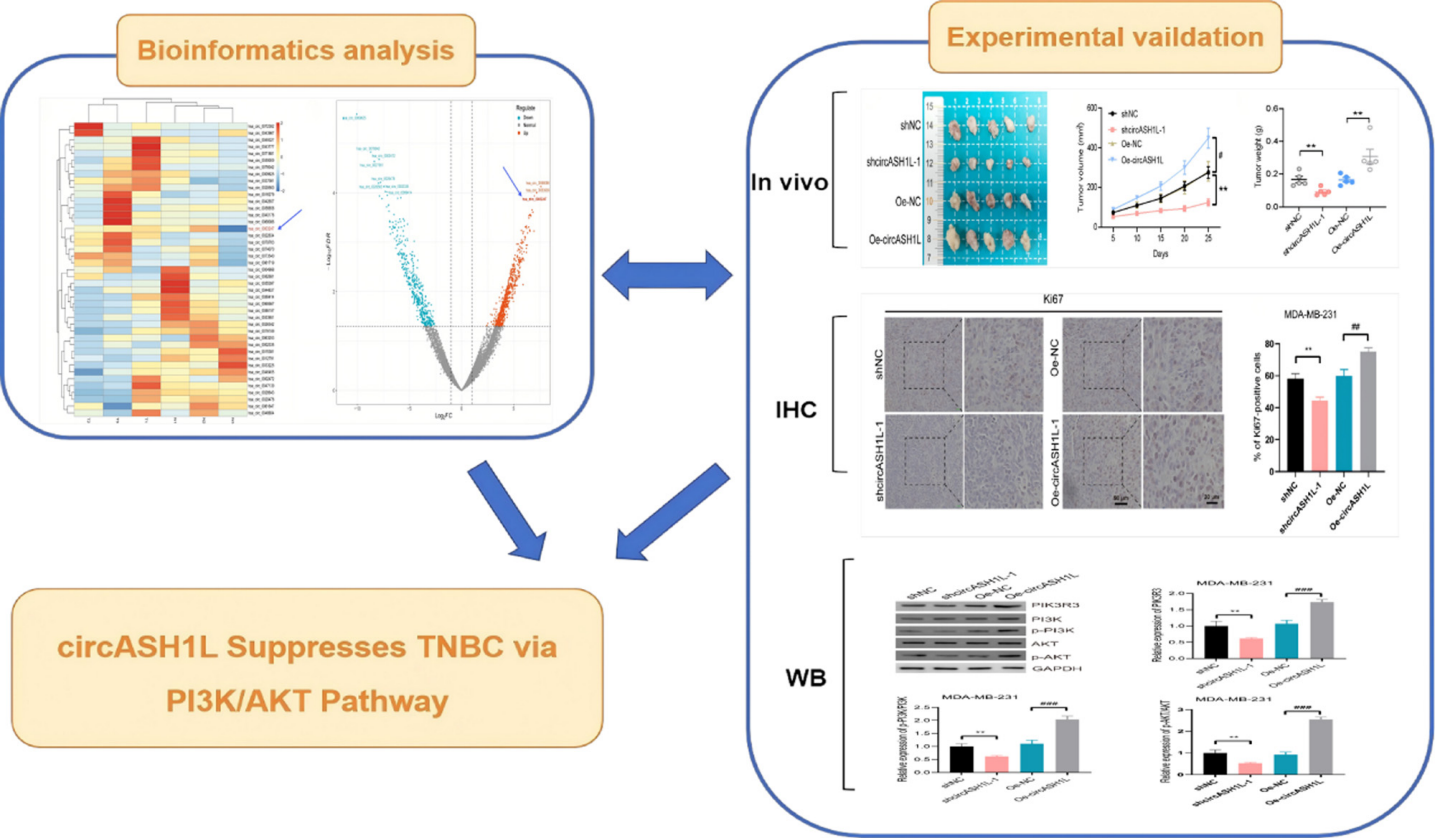
Flow chart of the mechanism study of circASH1L inhibiting TNBC via PI3K/AKT.

This study demonstrates that circASH1L is over-expressed in breast cancer, as verified by bioinformatics and animal experiments (Figure 1), and influences the growth of TNBC tumors in nude mice by modulating the PI3K/AKT pathway. The study’s bioinformatics analysis identified circASH1L as upregulated in breast cancer tissues, acting as a tumor promoter in TNBC. In animal experiments, circASH1L knockdown suppressed TNBC nude mouse tumor growth and cancer cell proliferation, whereas its overexpression produced the opposite outcome. circASH1L can regulate the growth of TNBC nude mouse tumors. CircASH1L knockdown suppressed PI3K/AKT signaling pathway activity, whereas its over-expression enhanced this pathway. circASH1L influences TNBC nude mouse subcutaneous tumor growth through the PI3K/AKT signaling pathway. In short, we have elucidated the regulatory mechanism of circASH1L on the growth of TNBC nude mouse subcutaneous tumors through the PI3K/AKT pathway, providing potential targets in the development of new pharmaceutical agents for therapeutic of TNBC.

## 2 Materials and methods

### 2.1 Bioinformatics analysis

The gene expression omnibus (GEO) database’s TNBC dataset (GSE113230) was chosen, comprising three TNBC samples and three normal breast cancer samples. The GEO2R tool from the GEO platform was utilized to identify relevant circular RNAs and analyze circASH1L expression in TNBC. The heat map and volcano map of high-expression and low-expression circRNAs were visualized based on R software. The screening criteria for differentially expressed lncRNAs were |log2(fold change)| > 1 and the corrected *P* value < 0.05.

### 2.2 Experimental procedures

#### 2.2.1 Animals selection and grouping

Twenty female BALB/C-nu nude mice were selected as research subjects, with an age of 6–7 weeks, an average age of 6.5 ± 1.02 weeks, a body weight of 15–20 g, and an average body weight of 17.21 ± 2.31 g. All animals were kept in a sterile environment with an ambient temperature of 22 ± 1°C and a relative humidity of 50 ± 10%. The dark and light periods were changed every 12 h. The animals were free to drink water and eat. The feed was LabDiet 5053. They were randomly divided into shNC group, shcircASH1L-1 group, Oe-NC group, and Oe-circASH1L group. This study excluded nude mice that did not meet the research requirements, such as failure of transplanted tumor modeling, and the treatment of animals was in full compliance with animal ethics requirements. The differences in weight, age, etc. between the groups did not influence the experimental conclusions (*P* > 0.05).

#### 2.2.2 Culture of human TNBC cell lines

The human TNBC cell line MBA-MD-231 was routinely cultured and divided into four treatment groups: shNC, shcircASH1L-1, Oe-NC, and Oe-circASH1L. The shNC group cells were transfected with non-targeted shRNA as the control group for shRNA treatment, the shcircASH1L-1 group was treated with shRNA targeted circASH1L-1 knockdown, the Oe-NC group was treated with blank vector introduction as the control of overexpression vector, and the Oe-circASH1L group was transfected with circASH1L overexpression vector to overexpress circASH1L.

#### 2.2.3 Construction of transplant tumor model

To establish a TNBC nude mouse transplant tumor model, four groups of MBA-MD-231 cell lines, post-treatment, were injected subcutaneously into the backs of four groups of nude mice (1 × 10^7^ cells/mL). The routine conditions of the nude mice, including food intake and metabolism, were documented, and the growth of the transplanted tumors was monitored. The transplanted tumor volumes were measured and documented every 3 days using the formula *V* = (length × width)^2/2^. The models in each group were killed by cervical dislocation 30 days after cell inoculation, and the weight of the transplanted tumors in each group was weighed. The tumor volume was measured by two professional technicians in a double-blind manner with a caliper accuracy of 0.01 mm. The intraclass correlation coefficient (ICC) was calculated for the measurement results, with ICC > 0.85 being the standard for good consistency.

#### 2.2.4 Immunohistochemistry method

Ki-67 immunohistochemistry was performed on tumor tissues from each group to assess cancer cell proliferation. Following antigen repair, tissue sections were treated with Ki-67 primary antibody, developed using an HRP-labeled secondary antibody and DAB, and counterstained with hematoxylin for the cell nuclei. The proportion of Ki-67 positive cells was observed and calculated under a microscope for quantitative image representation [19].

#### 2.2.5 Western blotting

Tumor tissues from each group were selected, and the extraction of total protein was performed using a protein extraction kit. The total protein underwent separation via SDS-PAGE electrophoresis and was then transferred onto a PVDF membrane. After blocking, the primary antibodies of PIK3R3 (Abcam, 27035-1-AP), PI3K (Abcam, ab191606), p-PI3K (Abcam, ab278733), AKT (Abcam, ab8805), p-AKT (Abcam, ab8932), and GAPDH (Abcam, ab22555) and the secondary antibodies labeled with HRP were incubated in turn. ECL staining was conducted with GAPDH serving as the internal reference. ImageJ software [20] was utilized to analyze and quantify the experimental results, and the experimental data were normalized relative to the control group (the control group was set as 1).

### 2.3 Statistical analysis

Using R software (4.2.1) and GraphPad Prism (9.0) to analyze all data. R was used for bioinformatics analysis, and packages such as pheatmap and ggplot2 were used. GraphPad Prism was used for other data analysis and visualization. Counting data such as tumor volume and proportion of ki-67 positive cells were expressed as “%” and subjected to ×2 test. Quantitative data, including tumor volume and relative expression levels in the western blot (WB) experiment, were represented as “mean ± SD” and analyzed using a *t*-test. The Mann–Whitney *U-*test or Kruskal–Wallis test was used for non-normally distributed data. Statistical significance was established with a *P* value of less than 0.05.


**Ethical approval:** The Ethics Committee of Jinan University approved this experiment (Ethical approval number: FSFY-MEC-2023-014).

## 3 Results

### 3.1 circASH1L is highly expressed in TNBC

**Figure 2:**
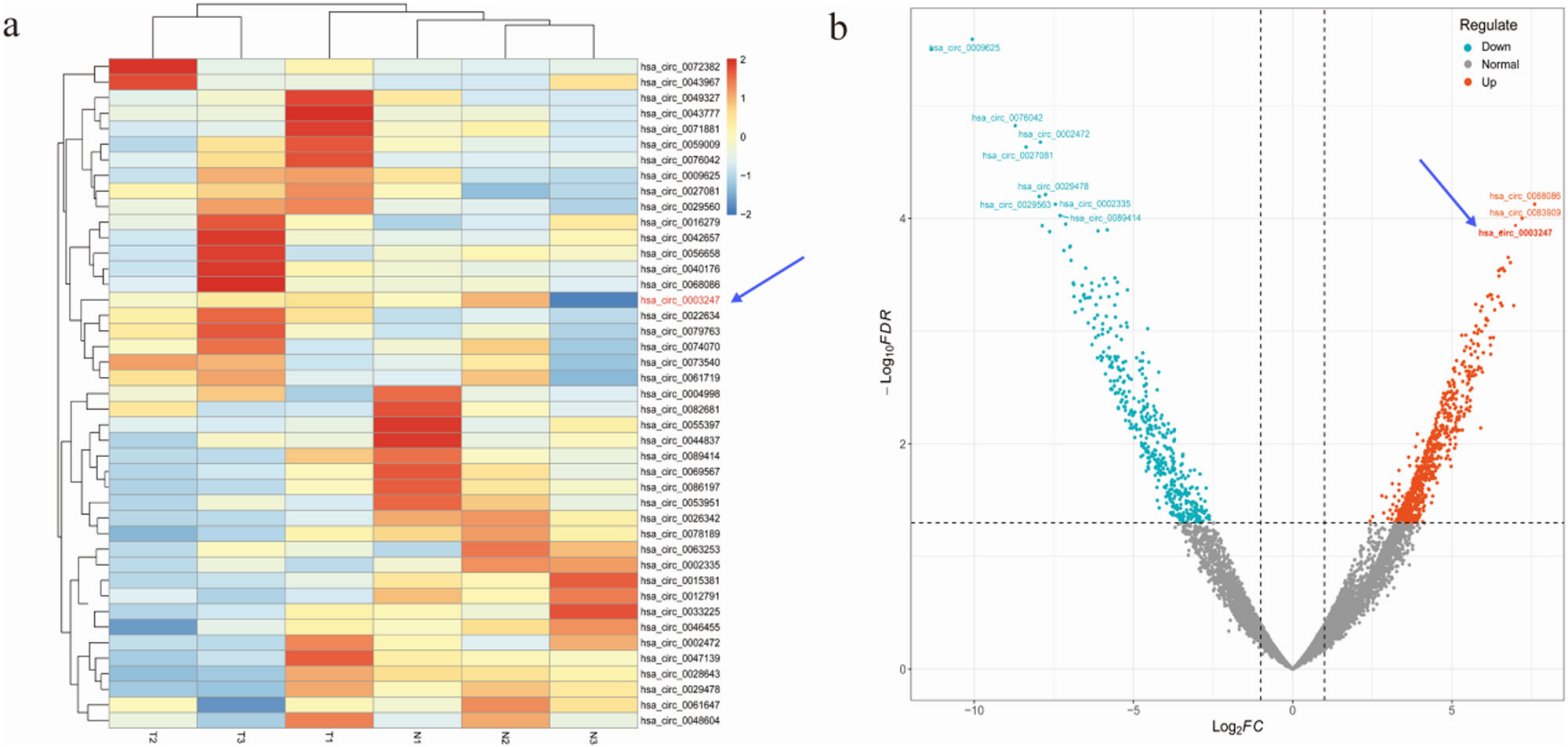
circASH1L is highly expressed in TNBC: (a) related circRNA heat map and (b) related circRNA volcano map. Data from GEO dataset GSE113230.

In order to distinguish the level difference of circASH1L in TNBC and normal tissues, we selected the GSE113230 dataset [containing TNBC samples (*n* = 3) and normal samples (*n* = 3)] in GEO database at the clinical level based on bioinformatics for analysis. The study identified 43 circRNAs significantly associated with TNBC, with hsa_circ_0003247 (circASH1L) showing notably higher expression in TNBC compared to the normal group (*P* < 0.05). The findings demonstrate that circASH1L is significantly overexpressed in the tumor samples from TNBC patients, serving as a tumor-promoting factor. Visualization of these results is provided in Figure 2.

### 3.2 circASH1L expression level significantly affects the growth of TNBC nude mouse tumors

**Figure 3:**
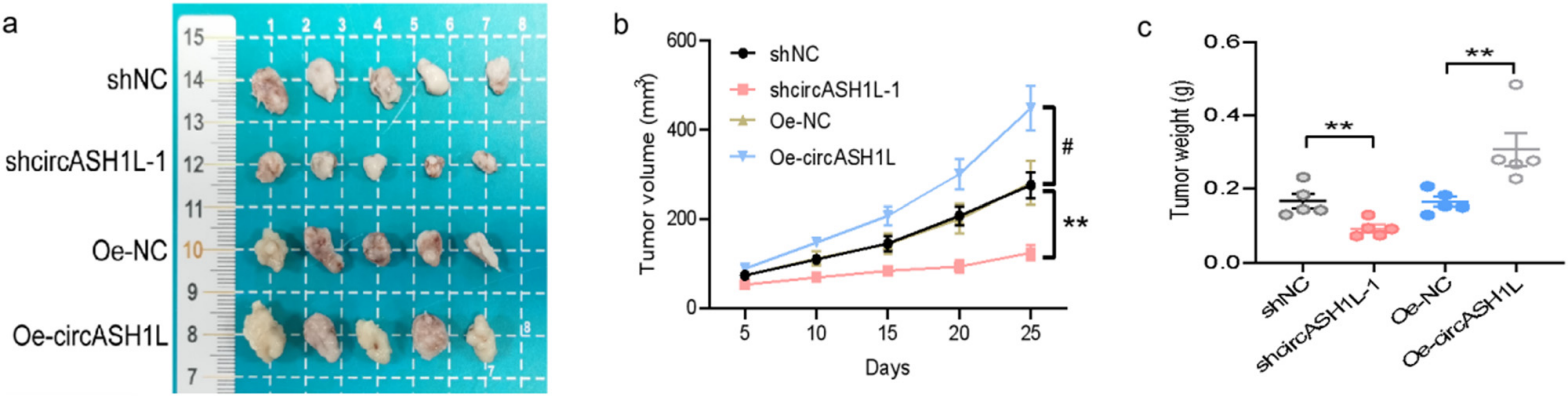
Significant impact of circASH1L expression levels on the growth of TNBC tumors in nude mice, as evidenced by a tumor image, a volume comparison line graph, and a tumor comparison scatter plot (^#^ *P* < 0.05, **P < 0.01). (a) Representative tumor images; (b) tumor volume growth curve; (c) tumor weight scatter plot.

To investigate how circASH1L expression influences tumor growth in TNBC *in vivo* models, human TNBC cells were cultured to generate TNBC xenograft models. At the same time, we knocked down and overexpressed circASH1L in the TNBC nude mouse model and analyzed the effect of circASH1L on the volume and mass of TNBC nude mice tumors. The study results demonstrated that the growth of tumors and nude mice in all four groups was favorable, and the modeling success rate was 100% (Figure 3a). Comparison of tumor volume (Figure 3b) and mass (Figure 3c) revealed no significant differences between the shNC and Oe-NC groups (*P* > 0.05). However, significantly smaller tumor volume and mass were observed in the shcircASH1L-1 group compared to the shNC group (*P* < 0.05), whereas the Oe-circASH1L group showed significantly greater values than the Oe-NC group (*P* < 0.05), indicating statistically significant differences. The findings indicated that circASH1L expression levels significantly influenced TNBC nude mice tumor growth. The growth of TNBC tumors in nude mice was positively correlated with circASH1L expression levels, as overexpression enhanced tumor growth while knockdown inhibited it.

### 3.3 circASH1L expression level affects the proliferation of TNBC nude mouse cancer cells

**Figure 4:**
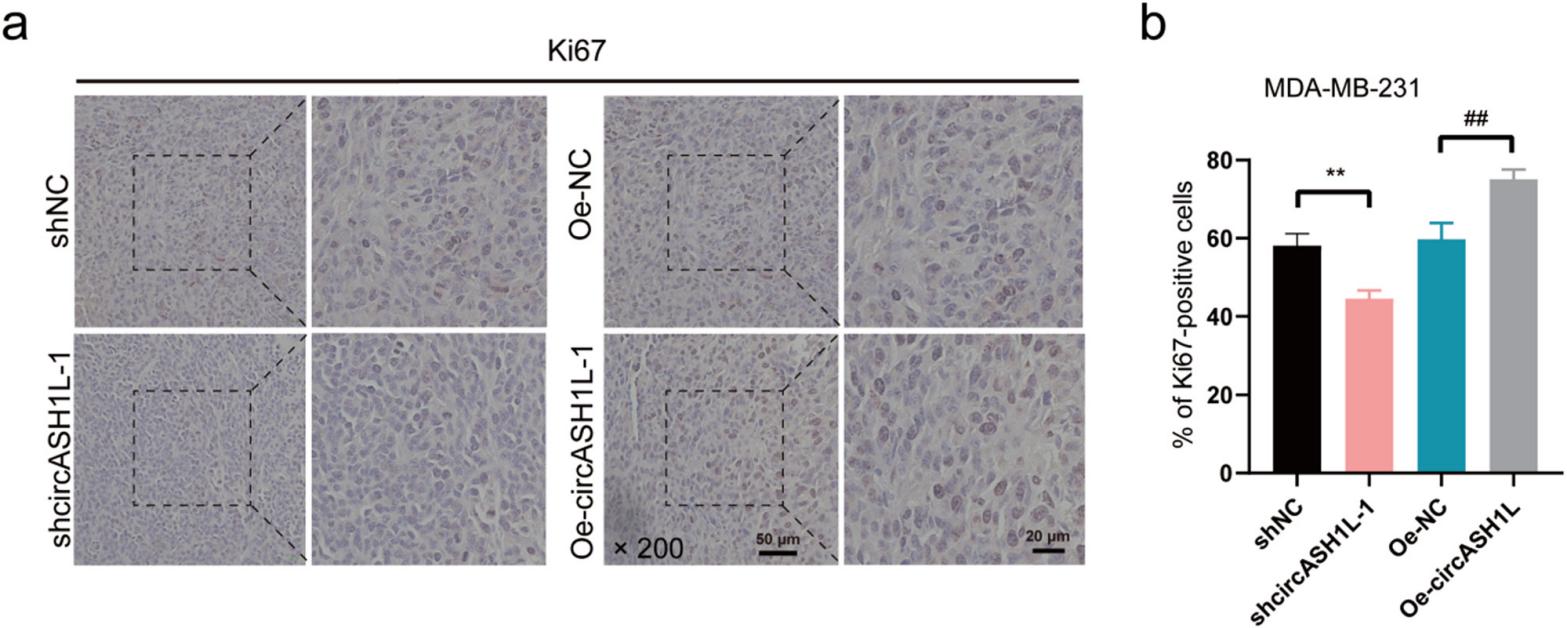
circASH1L expression level affects TNBC nude mouse cancer cell proliferation: (a) Ki67 expression diagram in four groups of nude mouse cancer tissues and (b) Ki67 expression positive rate comparison bar graph in four groups, ^##^ *P* < 0.01, **P < 0.01).

In order to analyze the effect of circASH1L level on the proliferation of TNBC nude mice cancer cells, TNBC nude mice models with circASH1L overexpression and knockdown were selected. To verify the marker protein Ki67 in TNBC cancer cells, immunohistochemical experiments were carried out. The findings indicated no significant difference in the Ki67 cell positive expression rate between the shNC and Oe-NC groups (*P* > 0.05). The Ki67 cell positive expression rate was significantly lower in the shcircASH1L-1 group than in the shNC group (*P* < 0.05). The Oe-circASH1L group exhibited a significantly higher positive expression rate of Ki67 cells compared to the Oe-NC group (*P* < 0.05). The study found that circASH1L expression notably influenced the proliferative behavior of TNBC cells in xenograft models of nude mice. CircASH1L overexpression and knockdown can promote and inhibit the proliferation of TNBC nude mouse cancer cells, respectively. That is, circASH1L showed a positive correlation trend in regulating the proliferation of TNBC nude mouse cancer cells (Figure 4).

### 3.4 circASH1L influences PI3K/AKT pathway marker protein expression in TNBC nude mice

**Figure 5:**
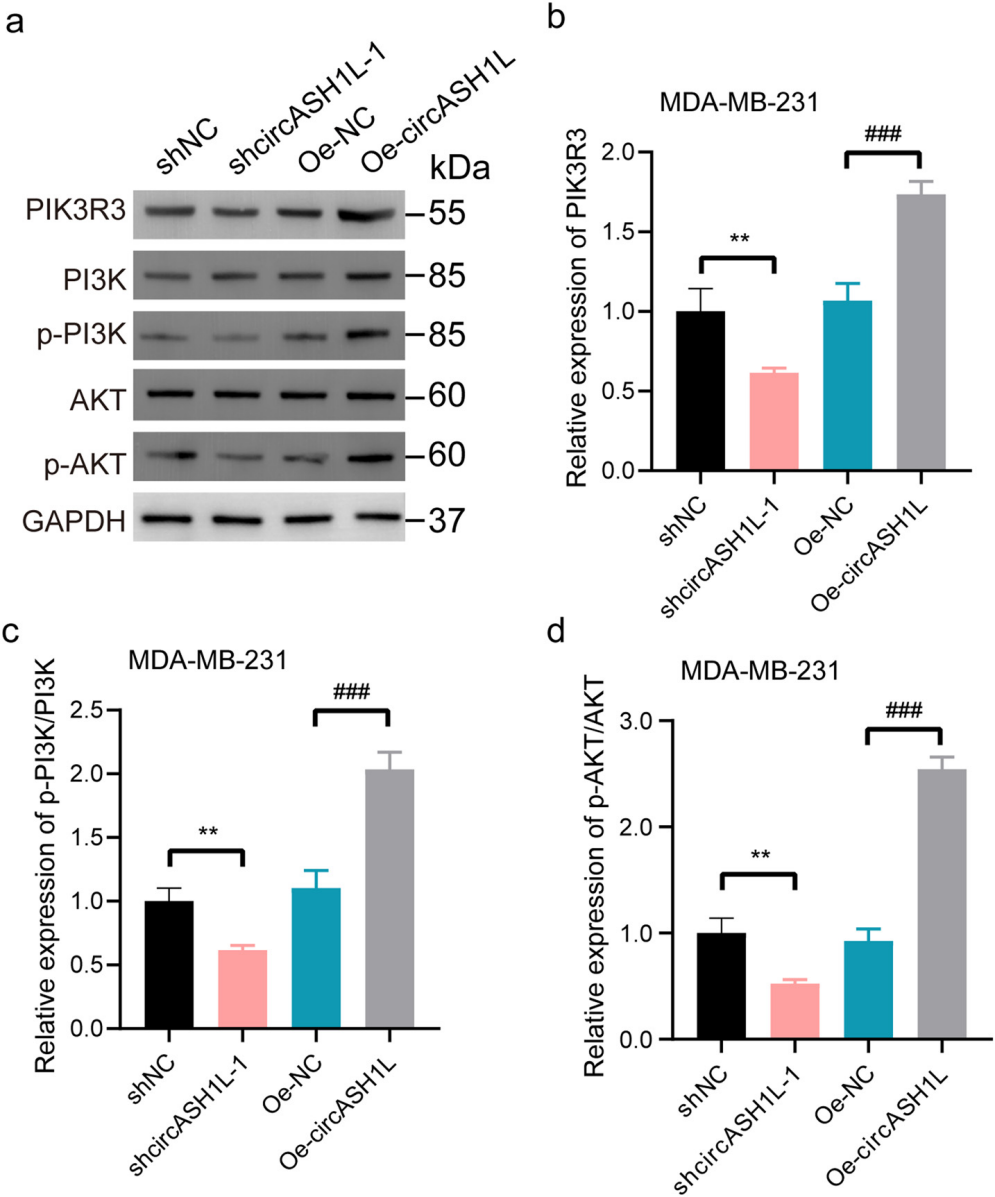
(a) Representative Western blot results for PIK3R3, PI3K, p-PI3K, AKT, and p-AKT. CircASH1L influences PI3K/AKT pathway marker protein expression in TNBC nude mice, as shown in a WB experimental results bar graph. Panels (b)–(d) compare the levels of PIK3R3, PI3K/p-PI3K, and AKT/p-AKT across four groups. **P < 0.01, ^###^ *P* < 0.001.

We employed western blot analysis to assess the impact of circASH1L knockdown and overexpression on the expression levels of PIK3R3, PI3K, p-PI3K, AKT, and p-AKT, key marker proteins of the PI3K/AKT pathway, in TNBC nude mice. The analysis indicated that the shcircASH1L-1 group exhibited significantly lower levels of PIK3R3, PI3K/p-PI3K, and AKT/p-AKT compared to the shNC group (*P* < 0.05). The Oe-circASH1L group showed significantly elevated levels of PIK3R3, PI3K/p-PI3K, and AKT/p-AKT compared to the Oe-NC group (*P* < 0.05). The statistically significant variations indicate that circASH1L expression levels significantly affect the PI3K/AKT signaling pathway activity in TNBC nude mice. The overexpression and knockdown of circASH1L enhance and suppress the regulatory function of the PI3K/AKT signaling pathway, respectively, mirroring the impact of circASH1L expression on tumor growth in TNBC nude mice (Figure 5).

## 4 Discussion

TNBC has the poorest prognosis and the fewest treatment options among breast cancer subtypes. Therefore, it is very important to find new effective diagnostic and therapeutic targets for TNBC. In addition to mRNA, the study of non-coding RNA regulating cancer has also become a hot topic in recent years, especially non-coding RNA participates in the occurrence of cancer by regulating each other or mRNA. Currently known non-coding RNA mainly includes lncRNA, miRNA, and circRNA. Although research on circRNA in cancer is relatively recent compared to non-coding RNAs like miRNA and lncRNA [21,22], numerous studies have established its role in regulating cancers, including TNBC [13–16]. This study utilized bioinformatics analysis on the GSE113230 dataset from GEO, identifying 43 circRNAs significantly linked to TNBC. Notably, circASH1L was upregulated, indicating its high expression in TNBC, consistent with its expression trend in cervical cancer [17]. Therefore, we conclude that at the bioinformatics level, circASH1L is a carcinogenic factor for TNBC.

In the animal experiment verification experiment, we selected shNC group, shcircASH1L-1 group, Oe-NC group, and Oe-circASH1L group for nude mouse tumor formation experiment. The shcircASH1L-1 group exhibited significantly reduced tumor volume and mass compared to the shNC group, whereas the Oe-circASH1L group showed a significantly increased tumor volume and mass relative to the Oe-NC group. This shows that circASH1L knockdown can inhibit the growth of TNBC nude mouse tumors, while circASH1L overexpression can promote the growth of TNBC nude mouse tumors. circASH1L acts as a carcinogenic factor in TNBC nude mice, aligning with both the bioinformatics analysis results of this study and the trends observed in cervical cancer animal experiments [17], suggesting that it may have a cross-cancer cancer-promoting function. In the Ki67 immunohistochemistry experiment, the shcircASH1L-1 group exhibited a significantly lower positive expression rate of Ki67 cells compared to the shNC group, while the Oe-circASH1L group showed a significantly higher rate than the Oe-NC group. This indicates that circASH1L knockdown reduces TNBC cell proliferation, whereas its overexpression enhances proliferation, corroborating the animal experiment results.

In the WB experiment, the shcircASH1L-1 group exhibited significantly reduced levels of PIK3R3, PI3K/p-PI3K, and AKT/p-AKT compared to the shNC group, while the Oe-circASH1L group showed significantly elevated levels of AKT and p-AKT relative to the Oe-NC group. This indicates that circASH1L influences the PI3K/AKT pathway marker proteins levels in TNBC nude mice. Current studies have confirmed that there are tumor PI3K/AKT pathway mutations in TNBC [23,24]. The PI3K/AKT pathway influences TNBC progression by modulating cancer cell growth and apoptosis [25]. The PI3K/AKT pathway is crucial in the treatment and drug resistance mechanisms of TNBC. Currently, circRNAs like circRPPH1, circTRIM1, and circPRKCI are known to influence the PI3K/AKT pathway, contributing to the regulatory mechanisms involved in the occurrence and progression of TNBC [26–28]. This study confirms the accuracy and credibility of circASH1L-1’s mechanism of action, as it similarly targets the PI3K/AKT signaling pathway to drive the progression of TNBC tumors.

This study demonstrates that circASH1L acts as a carcinogenic factor in TNBC, influencing tumor growth in TNBC nude mice and cancer cell proliferation via the PI3K/AKT pathway. However, due to limited working time, this study still has certain limitations, such as lack of further verification at the cellular and clinical levels, and focusing on only one signaling pathway. Future work will increase experimental verification at other levels and expand research on other signaling pathways.

## References

[1] Xu J, Sun J, Ho PY, Luo Z, Ma W, Zhao W, et al. Creatine based polymer for codelivery of bioengineered microRNA and chemodrugs against breast cancer lung metastasis. Biomaterials. 2019;210:25–40. 10.1016/j.biomaterials.2019.04.025 PMC6538300 31054369

[2] Tan DJY, Winnerdy FR, Lim KW, Phan AT. Coexistence of two quadruplex-duplex hybrids in the PIM1 gene. Nucleic Acids Res. 2020;48(19):11162–71. 10.1093/nar/gkaa752 PMC7641742 32976598

[3] Tariq A, Hao Q, Sun Q, Singh DK, Jadaliha M, Zhang Y, et al. LncRNA-mediated regulation of SOX9 expression in basal subtype breast cancer cells. RNA. 2020;26(2):175–85. 10.1261/rna.073254.119 PMC6961546 31690584

[4] Liu Y, Hu Y, Xue J, Li J, Yi J, Bu J, et al. Advances in immunotherapy for triple-negative breast cancer. Mol Cancer. 2023;22(1):145. 10.1186/s12943-023-01850-7 PMC10474743 37660039

[5] Ye F, Dewanjee S, Li Y, Jha NK, Chen ZS, Kumar A, et al. Advancements in clinical aspects of targeted therapy and immunotherapy in breast cancer. Mol Cancer. 2023;22(1):105. 10.1186/s12943-023-01805-y PMC10324146 37415164

[6] Adrada BE, Moseley TW, Kapoor MM, Scoggins ME, Patel MM, Perez F, et al. Triple-negative breast cancer: histopathologic features, genomics, and treatment. Radiographics. 2023;43(10):e230034. 10.1148/rg.230034 PMC10560981 37792593

[7] Mann GB, Skandarajah AR, Zdenkowski N, Hughes J, Park A, Petrie D, et al. Postoperative radiotherapy omission in selected patients with early breast cancer following preoperative breast MRI (PROSPECT): primary results of a prospective two-arm study. Lancet. 2024;403(10423):261–70. 10.1016/S0140-6736(23)02476-5 38065194

[8] Zhang Y, Fan J, Wang X, Wu Z, Ma W, Ma B. Role of ICAM-1 in triple-negative breast cancer. Open Med. 2024;19(1):20240969. 10.1515/med-2024-0969 PMC11117456 38799250

[9] Wu S, Lu J, Zhu H, Wu F, Mo Y, Xie L, et al. A novel axis of circKIF4A-miR-637-STAT3 promotes brain metastasis in triple-negative breast cancer. Cancer Lett. 2024;581:216508. 10.1016/j.canlet.2023.216508 38029538

[10] Xu J, Wu KJ, Jia QJ, Ding XF. Roles of miRNA and lncRNA in triple-negative breast cancer. J Zhejiang Univ Sci B. 2020;21(9):673–89. 10.1631/jzus.B1900709 PMC7519626 32893525

[11] Wang Z, Li Y, Yang J, Liang Y, Wang X, Zhang N, et al. Circ-TRIO promotes TNBC progression by regulating the miR-432-5p/CCDC58 axis. Cell Death Dis. 2022;13(9):776. 10.1038/s41419-022-05216-7 PMC9458743 36075896

[12] Lan J, Wang L, Cao J, Wan Y, Zhou Y. circBRAF promotes the progression of triple-negative breast cancer through modulating methylation by recruiting KDM4B to histone H3K9me3 and IGF2BP3 to mRNA. Am J Cancer Res. 2024;14(5):2020–36. 10.62347/OOLG5765 PMC11162659 38859856

[13] Zheng X, Huang M, Xing L, Yang R, Wang X, Jiang R, et al. The circRNA circSEPT9 mediated by E2F1 and EIF4A3 facilitates the carcinogenesis and development of triple-negative breast cancer. Mol Cancer. 2020;19(1):73. 10.1186/s12943-020-01183-9 PMC7137343 32264877

[14] Zheng SR, Huang QD, Zheng ZH, Zhang ZT, Guo GL. circGFRA1 affects the sensitivity of triple-negative breast cancer cells to paclitaxel via the miR-361-5p/TLR4 pathway. J Biochem. 2021;169(5):601–11. 10.1093/jb/mvaa148 33481008

[15] He R, Liu P, Xie X, Zhou Y, Liao Q, Xiong W, et al. circGFRA1 and GFRA1 act as ceRNAs in triple negative breast cancer by regulating miR-34a. J Exp Clin Cancer Res. 2017;36(1):145. 10.1186/s13046-017-0614-1 PMC5644184 29037220

[16] Li J, Gao X, Zhang Z, Lai Y, Lin X, Lin B, et al. CircCD44 plays oncogenic roles in triple-negative breast cancer by modulating the miR-502-5p/KRAS and IGF2BP2/Myc axes. Mol Cancer. 2021;20(1):138. 10.1186/s12943-021-01444-1 PMC8543802 34696797

[17] Zhang J, Zhang Y, Li X, Bao Y, Yang J. Has_circ_ASH1L acts as a sponge for miR-1254 to promote the malignant progression of cervical cancer by targeting CD36. Cancer Gene Ther. 2025;32(2):214–26. 10.1038/s41417-024-00866-5 39748122

[18] He J, Fu H, Li C, Deng Z, Chang H. Eriodictyol inhibits breast carcinogenesis by targeting circ_0007503 and repressing the PI3K/Akt pathway. Phytomedicine. 2022;102(7):154159. 10.1016/j.phymed.2022.154159 35580441

[19] Badr NM, Zaakouk M, Zhang Q, Kearns D, Kong A, Shaaban AM. Concordance between ER, PR, Ki67, and HER2-low expression in breast cancer by MammaTyper RT-qPCR and immunohistochemistry: implications for the practising pathologist. Histopathology. 2024;85(3):437–50. 10.1111/his.15193 38651302

[20] Cheng S, Wan X, Yang L, Qin Y, Chen S, Liu Y, et al. RGCC-mediated PLK1 activity drives breast cancer lung metastasis by phosphorylating AMPKα2 to activate oxidative phosphorylation and fatty acid oxidation. J Exp Clin Cancer Res. 2023;42(1):342. 10.1186/s13046-023-02928-2 PMC10722681 38102722

[21] Nemeth K, Bayraktar R, Ferracin M, Calin GA. Non-coding RNAs in disease: from mechanisms to therapeutics. Nat Rev Genet. 2024;25(3):211–32. 10.1038/s41576-023-00662-1 37968332

[22] Wei L, Sun J, Zhang N, Zheng Y, Wang X, Lv L, et al. Noncoding RNAs in gastric cancer: implications for drug resistance. Mol Cancer. 2020;19(1):62. 10.1186/s12943-020-01185-7 PMC7081551 32192494

[23] Eustace AJ, Lee MJ, Colley G, Roban J, Downing T, Buchanan PJ. Aberrant calcium signalling downstream of mutations in TP53 and the PI3K/AKT pathway genes promotes disease progression and therapy resistance in triple negative breast cancer. Cancer Drug Resist. 2022;5(3):560–76. 10.20517/cdr.2022.41 PMC9511797 36176752

[24] Shrivastava S, Kulkarni P, Thummuri D, Jeengar MK, Naidu VG, Alvala M, et al. Piperlongumine, an alkaloid causes inhibition of PI3K/Akt/mTOR signaling axis to induce caspase-dependent apoptosis in human triple-negative breast cancer cells. Apoptosis. 2014;19(7):1148–64. 10.1007/s10495-014-0991-2 24729100

[25] Hakeem AN, El-Kersh DM, Hammam O, Elhosseiny A, Zaki A, Kamel K, et al. Piperine enhances doxorubicin sensitivity in triple-negative breast cancer by targeting the PI3K/Akt/mTOR pathway and cancer stem cells. Sci Rep. 2024;14(1):18181. 10.1038/s41598-024-65508-0 PMC11303729 39107323

[26] Zhang C, Yu Z, Yang S, Liu Y, Song J, Mao J, et al. ZNF460-mediated circRPPH1 promotes TNBC progression through ITGA5-induced FAK/PI3K/AKT activation in a ceRNA manner. Mol Cancer. 2024;23(1):33. 10.1186/s12943-024-01944-w PMC10865535 38355583

[27] Li Y, Wang Z, Yang J, Sun Y, He Y, Wang Y, et al. CircTRIM1 encodes TRIM1-269aa to promote chemoresistance and metastasis of TNBC via enhancing CaM-dependent MARCKS translocation and PI3K/AKT/mTOR activation. Mol Cancer. 2024;23(1):102. 10.1186/s12943-024-02019-6 PMC11097450 38755678

[28] Wang X, Song H, Fang L, Wu T. EIF4A3-mediated circPRKCI expression promotes triple-negative breast cancer progression by regulating WBP2 and PI3K/AKT signaling pathway. Cell Death Discov. 2022;8(1):92. 10.1038/s41420-022-00892-y PMC8891274 35236829

